# Nocardia – Opportunistic chest infection in elderly: A case report

**DOI:** 10.1186/1757-1626-1-122

**Published:** 2008-08-21

**Authors:** Kaushik Sanyal, Kanagasabesan Sabanathan

**Affiliations:** 1 Department of Medicine, Norfolk and Norwich University Hospital, Norwich, UK

## Abstract

In this rare case a non-immunocompromised patient with old Tuberculosis on low dose of steroids presents with opportunistic infection of a weakly aerobic gram positive acid fast, filamentous bacteria called Nocardia.

An 80 year old non-smoking white female presented with cough, shortness of breath and purulent sputum.

Initial antibiotics given were not helpful. Later microbial diagnosis was Nocardia in sputum sample which was uncommon in a non-immunocompromised. She responded to co-trimoxazole therapy.

## Background

Nocardia organism is filamentous gram positive rods. They are aerobic. Human pathogens infect by inhalation of airborne bacilli or the traumatic inoculation of organism into skin. Overall 80% present as invasive pulmonary infection, disseminated disease abscess; 20% present as cellulitis. Debilitated patients have a 45% mortality rate even with appropriate therapy.

## Case presentation

**Figure 1:**
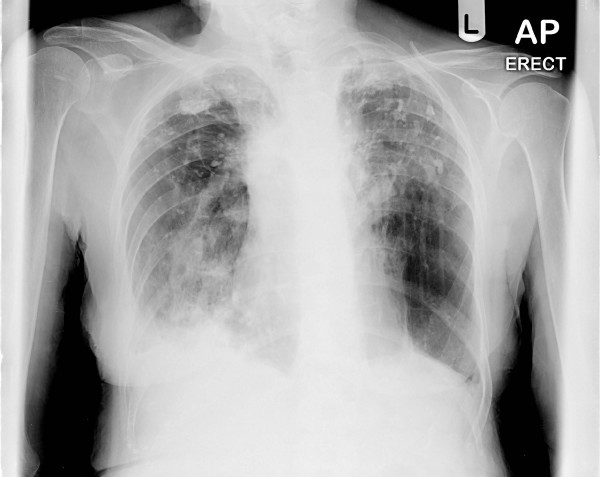
Chest X-ray showing consolidation and bronchiectatic changes.

**Figure 2:**
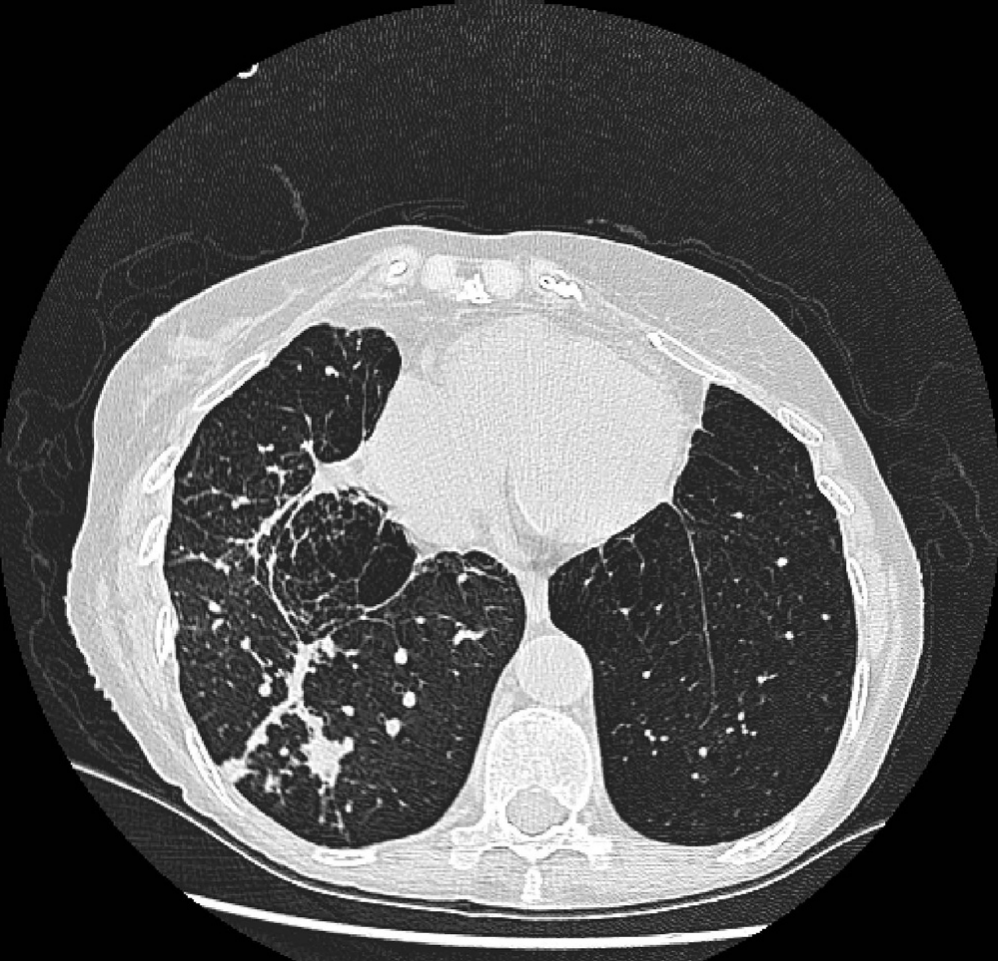
C**T scan showing bronchiectatic cavity of old tuberculosis which grew Nocardia.**

An 80 year old non-smoker white female was admitted with shortness of breath and cough with purulent sputum. Relevant past history of polymyalgia rheumatica, for which she was on regular low dose steroids. She also informed past history of Tuberculosis (in 1951) treated in sanatorium. She was hypoxic with oxygen saturation 79.9% on air. Her inflammatory markers were high. Chest X-ray [Fig 1] and Computed tomographic scan [Fig 2] revealed old calcification of right apical lobe and consolidation and bronchiectatic changes of right lower lobe. She was treated with bronchodilators and antibiotics. She was still hypoxic with high inflammatory markers. The microbial diagnosis was established after isolating Nocardia in sputum sample. Subsequently she was subsequently treated with trimethoprim and sulphamethoxazole for 6 months and offered further follow-up. Further follow-up clinic we found out that she responded to the treatment.

## Discussion

In this report, we present a rare case of a non-immunocompromised patient with old Tuberculosis but she has been on low dose of steroids. Nocardia is a weakly aerobic gram positive acid fast, filamentous bacteria. They are an important opportunistic infection in elderly and immunocompromised affecting lung, brain and skin. Nocardiasis is sporadic and have higher incidence in the immunocompromised population. There is no age, race predilection [1]. Pre-existing lung condition, in this of case of tuberculosis increase the risk of contracting the infection. Bronchiectasis is an important risk factor of Nocardia colonisation. The presentations are cough, fever and difficulties in breathing. The spread is sporadic, and are usually found in dust and soil. The use of Ziehl Neelson technique with a weaker acid concentration can result in identification of a variety of acid fast organisms [2]. Generalised infection involves cutaneous joint and pulmonary cause. Further dissemination involves acute and chronic symptoms. There are no person to person transmission. Core diagnostic test include sputum culture and bronchoscopy.

Standard therapy is trimethoprim-sulphamethoxazole for 6 months to 1 year. Refractory case needs imipenem and amikacin. Nocardiais should be suspected in immunosuppressed patients.

## Conclusion

Pulmonary bronchiectasis is difficult to diagnose, which delays its diagnosis and a high level of suspicion is required in patients with underlying chronic condition or chronic steroid use [3]. Nocardia when involves the central nervous system leads to a poor prognosis, implies an early diagnosis and prompt treatment. Death occurs from sepsis, overwhelming pneumonia or brain abscess. Mortality is increased in disseminated disease involving 2 or more organs [4]. Mortality is more in patients on corticosteroids or chemotherapy. Nocardiosis should be an important differential of any chronic pneumonia not responding to the antibiotic treatment.

## Competing interests

The authors declare that they have no competing interests.

## Authors' contributions

KS carried out literature search. KS participated in the sequence alignment and draft of manuscript. All authors read and approved the final manuscript
